# A taxonomic revision of the genus *Angelica* (Apiaceae) in Taiwan with a new species *A. aliensis*

**DOI:** 10.1186/s40529-023-00407-7

**Published:** 2024-01-22

**Authors:** Jenn-Che Wang, Hung-Hsin Chen, Tsai-Wen Hsu, Kuo-Hsiang Hung, Chi-Chun Huang

**Affiliations:** 1 Department of Life Science, National Taiwan Normal University, Taipei, 106 Taiwan; 2 Wild Plants Division, Taiwan Biodiversity Research Institute, Nantou, 552 Taiwan; 3 Graduate Institute of Bioresources, Pingtung University of Science and Technology, Pingtung, 912 Taiwan; 4 Forestry and Biodiversity Research Center, National Pingtung University of Science and Technology, Pingtung, 912 Taiwan

**Keywords:** *Angelica*, cpDNA, nrDNA, Taxonomic revision, Taiwan

## Abstract

**Background:**

*Angelica* L. sensu lato is a taxonomically complex genus, and many studies have utilized morphological and molecular features to resolve its classification issues. In Taiwan, there are six taxa within *Angelica*, and their taxonomic treatments have been a subject of controversy. In this study, we conducted a comprehensive analysis incorporating morphological and molecular (cpDNA and nrDNA) characteristics to revise the taxonomic treatments of *Angelica* in Taiwan.

**Results:**

As a result of our research, we have revised the classification between *A. dahurica* var. *formosana* and *A. pubescens* and merged two varieties of *A. morrisonicola* into a single taxon. A new taxon, *A. aliensis*, has been identified and found to share a close relationship with *A. tarokoensis*. Based on the morphological and molecular characteristics data, it has been determined that the former three taxa should be grouped into the Eurasian *Angelica* clade, while the remaining four taxa should belong to the littoral *Angelica* clade. Furthermore, *Angelica* species in Taiwan distributed at higher altitudes displayed higher genetic diversity, implying that the central mountain range of Taiwan serves as a significant reservoir of plant biodiversity. Genetic drift, such as bottlenecks, has been identified as a potential factor leading to the fixation or reduction of genetic diversity of populations in most *Angelica* species. We provide key to taxa, synopsis, phenology, and distribution for each taxon of Taiwan.

**Conclusions:**

Our comprehensive analysis of morphological and molecular features has shed light on the taxonomic complexities within *Angelica* in Taiwan, resolving taxonomic issues and providing valuable insights into the phylogenetic relationships of *Angelica* in Taiwan.

**Supplementary Information:**

The online version contains supplementary material available at 10.1186/s40529-023-00407-7.

## Background

*Angelica* L. sensu lato (Apiaceae subfamily Apioideae) is a taxonomically complex and controversial group that encompasses many species, estimated to number approximately 110 (Wang et al. 2021; Liao et al. 2022). Taxa of *Angelica* are mainly distributed in the northern hemisphere, particularly in East Asia, where the highest number of species (approximately 55) can be found (Sheh et al. 2005; Wang et al. 2021). Some *Angelica* species have been used in traditional Chinese medicines and have great economic value (Sheh et al. 2005; Taiwan herbal pharmacopeia 3rd edition Committee 2019; Wang et al. 2021). Previous studies have used molecular and morphological data to ascertain evolutionary relationships and species boundaries (Liao et al. 2012, 2013, 2022; Wang et al. 2021). According to Liao et al. (2013), the majority of *Angelica* species can be classified into two main clades, the *Ostericum* clade and the *Angelica* group. The *Angelica* group consists of five major lineages: *Angelica* s. s., *Archangelica*, *Coelopeurum*, *Glehnia*, and littoral *Angelica* clades. Liao et al. (2022) further expanded on this classification by collecting approximately 100 taxa of *Angelica* and redefined the *Angelica* group into four lineages: the Eurasian *Angelica*, North American *Angelica*, *Archangelica* and littoral *Angelica* clades. In Taiwan, six taxa occur within *A. dahurica* (Fisch.) Benth. & Hook. var. *formosana* (Boiss.) Yen., *A. hirsutiflora* Liu, Chao & Chuang, *A. morii* Hayata, *A. morrisonicola* Hayata, *A. morrisonicola* var. nanhutashanensis Liu, Chao & Chuang, and *A. tarokoensis* Hayata (Kao 1993). *Angelica dahurica* var. *formosana* is distributed at low altitudes, and *A. hirsutiflora* is predominantly distributed in coastal areas in northern Taiwan. In contrast, *A. morii*, *A. morrisonicola*, and *A. morrisonicola* var. *nanhutashanensis* are distributed at high altitudes of over 3000 m in central Taiwan. *Angelica tarokoensis* is distributed at medium altitudes of approximately 400–2000 m in eastern and southern Taiwan. Except for *A. dahurica* var. *formosana*, which is considered the possible original species of the traditional Chinese drug “BaiZhi” (Liang et al. 2018), others are endemic and belong to the littoral *Angelica* clade (Kao 1993; Liao et al. 2022). Chen (2008) reported one new record taxon (*A. pubescens* Maxim) distributed at high altitudes in central Taiwan and one new taxon (*A. aliensis* H. H. Chen & J. C. Wang) only distributed at medium altitudes in southern Taiwan and treated *A. morrisonicola* var. *nanhutashanensis* as a synonym of *A. morrisonicola* based on morphology, pollen and distribution data. Morphological similarity has been attributed to the controversy concerning the taxonomic treatments of *Angelica* in Taiwan. *Angelica dahurica* var. *formosana*, and *A. pubescens* exhibit morphological similarities, but they inhabit different habitats. *Angelica morrisonicola* and *A. morrisonicola* var. *nanhutashanensis* were taxonomically separated based on the presence or absence of leaf surface hairs. Similarly, *A. aliensis* and *A. tarokoensis* were taxonomically differentiated based on the presence or absence of the cartilaginous margins of leaflets. Further studies are recommended to clarify the phylogenetic relationships of *Angelica* in Taiwan.

Species delimitation and the understanding of patterns of genetic variation are crucial aspects of biodiversity conservation (Hosegood et al. 2020; Chen et al. 2023). Identifying genetically distinct populations or evolutionary lineages within a species provides vital information for developing effective conservation strategies. Habitat destruction and overexploitation have been attributed to the endangerment of *Angelica* species. *Angelica dahurica* var. *formosana*, *A. hirsutiflora*, and *A. tarokoensis* are categorized as threatened species because of human overexploitation and habitat destruction (Editorial Committee of the Red List of Taiwan Plants 2017). Thus, appropriate conservation strategies are urgently needed for *Angelica* species in Taiwan.

The classification of *Angelica* in Taiwan is primarily based on morphological characteristics, such as plant size, leaf shape, presence or absence of trichomes, and habitat preferences, which are often used to distinguish different taxa within *Angelica* (Kao 1993). Morphological similarity among *Angelica* species makes it possible to establish plausible taxonomic relationships and classify them into distinct groups or species. The morphological characteristics alone may not always provide a complete understanding of evolutionary relationships (Sun and Downie 2010; Lei et al. 2022). Molecular evidence can also help to robustly validate and refine the phylogeny of *Angelica* in Taiwan. Molecular markers have been widely used to elucidate the phylogeny and systematics of plants (Huang et al. 2001, 2011; Chiang et al. 2006). Uniparental and biparental markers provide better resolution in plant phylogeny (Birky 1995; Huang et al. 2011). Nuclear ribosomal DNA internal transcribed spacer (ITS), external transcribed spacer (ETS) and chloroplast DNA have been widely applied to infer the phylogeny of *Angelica* (Feng et al. 2009; Liao et al. 2012, 2013, 2022; Wang et al. 2021). Previous studies have emphasized the phylogeny of the *Angelica *sensu stricto group, and some have focused on the littoral *Angelica* clade in Taiwan. In this study, a comprehensive approach combining morphological and molecular data was employed to investigate the taxonomic statements of *Angelica* in Taiwan. This integrative approach allows for a more comprehensive and accurate assessment of the phylogeny of *Angelica* species, shedding light on their evolutionary history and genetic relationships in the context of the local flora.

## Materials and methods

### Morphology

In this study, wild living plants of *Angelica* in Taiwan were examined. We also examined herbarium specimens from Herbarium, Research Center for Biodiversity, Academia Sinica, Taipei (HAST), Herbarium, National Taiwan University (TAI), Herbarium of Endemic Species Research Institute (TAIE), Herbarium, Taiwan Forestry Research Institute (TAIF), Herbarium, National Taiwan Normal University (TNU) and TNM Herbarium, Department of Botany, National Museum of Natural Science (TNM) in Taiwan to compare the morphological characteristics. By analyzing these living plants and the herbarium specimens, we aimed to assess the variations and similarities in morphological characteristics among different *Angelica* species in Taiwan (Table 1). The morphological characteristics, including leaf and stem surface, trichome type, bracteole type and number, reticulate veinlet, cartilaginous margins of leaflets, ternate pinnae decurrent and color of anther, were applied to distinguish these taxa. For *A. aliensis*, pollen grains from fresh samples underwent a dehydration process involving sequential immersion in ethanol solutions with concentrations of 75%, 85%, 95%, and 99.5%, followed by 100% acetone. After dehydration, the specimens were subjected to gold coating and subsequently observed using a JEOL JSM-5600 scanning electron microscope (JEOL Ltd., Tokyo, Japan).

**Table 1:** Morphological comparison of *Angelica* in Taiwan

Species	*A. aliensis*	*A. dahurica* var.* formosana*	*A. hirsutiflora*	*A. morii*	*A. morrisonicola*	*A. pubescens*	*A. tarokoensis*
Leaf surface	Glabrous	Glabrous	Glabrous	Glabrous	Hispidulous	Pubescent	Hispidulous
Stem surface	Glabrous	Hispidulous	Hispidulous	Glabrous	Pubescent	Pubescent	Glabrous
Reticulate veinlets	Prominent	Sunken	Sunken	Sunken	Sunken	Prominent	Prominent
Trichomes type	Glabrous	Hispid	Hispid	Glabrous	Velutinous	Velutinous	Hispid
Bracteole type	Narrowly-triangular	Linear	Elliptic	Linear	Linear	Linear	Narrowly-triangular
Bracteole number	4–6	8–10	10	0–4	10	0–4	10
Cartilaginous margins of leaflets	Absence	Absence	Absence	Absence	Absence	Absence	Presence
Ternate pinnae decurrent	Absence	Presence	Absence	Absence	Absence	Absence	Absence
Anther color	Purple	Yellow	Yellow	Purple	Yellow	Purple/yellow	Yellow
Vitta in the interval	1	1	1	1	1	3	1
Vitta in the commissure	2	2	4	2	2	8	2

### Plant materials

**Fig. 1:**
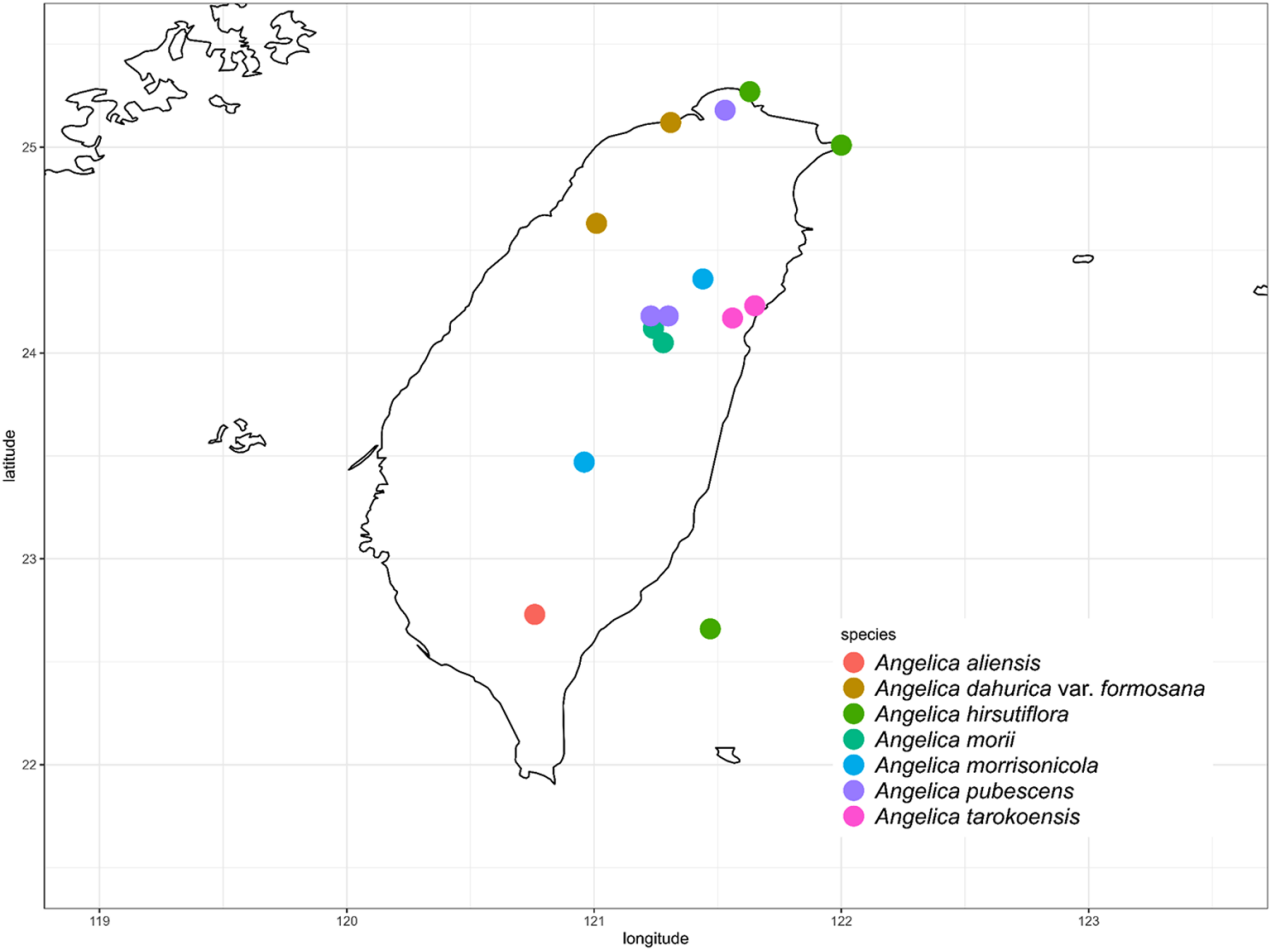
The sample locations of *Angelica* in Taiwan

We followed the treatments of Kao (1993) and Chen (2008) to classify the species used in this study. Seven taxa of *Angelica* in Taiwan, including 114 individuals, were sampled (Fig. 1, Table 2). Each taxon was collected from approximately 1–12 individuals according to population size. Few samples of *A. aliensis* and *A. tarokoensis* were collected due to their small population sizes in the wild. Young leaves were collected from the field and quickly dried using silica gels. The specimens of *A. dahurica* from China (Collector No.: Yu-Tang Zhao 465, Quanru Liu 01-1-098, and Feng Wu 2010036) in the TNM herbarium were also collected to extract the DNA. All samples were stored at − 80 ℃ until laboratory work.

**Table 2:** Sample location, code, coordination, size, nucleotide, nucleotide diversity (π), haplotype, haplotype diversity (Hd), and Tajima’s D of *Angelica* in Taiwan

Species	Location	Code	Longitude	Latitude	Sample size	nrDNA					cpDNA				
						Accession number	π	Haplotype	Hd	Tajima’s D	Accession number	π	Haplotype	Hd	Tajima's D
*Angelica aliensis*					3		0	1	0	–		0	1	0	-
	Wutai	WT	120.76	22.73	3	OR242589	0	1	0		OR240204	0	1	0	
*Angelica dahurica* var.* formosana*					20		0	1	0	–		0	1	0	-
	Linkou	LK	121.31	25.12	8	OR242587	0	1	0		OR240202	0	1	0	
	Nanzhuang	NZ	121.01	24.63	12	OR242587	0	1	0		OR240202	0	1	0	
*Angelica hirsutiflora*					34		0	1	0	–		0	1	0	-
	Magang	MG	122.00	25.01	12	OR242586	0	1	0		OR240201	0	1	0	
	Jinshan	JS	121.63	25.27	12	OR242586	0	1	0		OR240201	0	1	0	
	Green Island	GI	121.47	22.66	10	OR242586	0	1	0		OR240201	0	1	0	
*Angelica morii*					18		0	1	0	–		0.00193	2	0.523	2.221387*
	Hehuan	HH	121.24	24.12	10	OR242590	0	1	0		OR240206	0	1	0	
	Tianchi	TC	121.28	24.05	8	OR242590	0	1	0		OR240209	0	1	0	
*Angelica morrisonicola*					15		0	1	0	–		0	1	0	-
	Yushan	YS	120.96	23.47	5	OR242595	0	1	0		OR240210	0	1	0	
	Nanhutashan	NH	121.45	24.37	10	OR242595	0	1	0		OR240210	0	1	0	
*Angelica pubescens*					19		0	1	0	–		0.00129	2	0.526	2.00656*
	Datun	DT	121.53	25.18	10	OR242588	0	1	0		OR240203	0	1	0	
	Dayuling	DY	121.30	24.18	3	OR242588	0	1	0		OR240205	0	1	0	
	Fushoushan	FS	121.23	24.18	6	OR242588	0	1	0		OR240205	0	1	0	
*Angelica tarokoensis*					5		0.00063	2	0.4	-0.8165		0.00098	2	0.4	-0.973
	Qingshuishan	QS	121.65	24.23	1	OR242591	0	1	0		OR240208	0	1	0	
	Zhuilu	ZL	121.56	24.17	4	OR242594	0	1	0		OR240207	0	1	0	

* indicate *P* < 0.05

### DNA extraction, PCR amplification and sequencing

Genomic DNA was extracted from powdered tissues following the CTAB procedure (Murray and Thompson 1980). PCR amplifications of the *rps16-trnK* intergenic spacer of cpDNA (Liao et al. 2012) and the ITS region of nrDNA (Chiang et al. 2001) were performed in a 50 μL reaction using 10 ng template DNA, 25 μL GoTaq® Green Master Mix (Promega, Madison, WI, USA), and 5 pmol of each primer. PCRs were performed in a PCR thermal cycler using the following profile: an initial 10 min denaturation at 94 ℃, 30 cycles of 45 s denaturation at 94 ℃, 1 min 15 s annealing at 55 ℃ for two markers, and 1 min 30 s extension at 72 ℃, followed by a 10 min final extension at 72 ℃. All PCR products were purified and then sequenced directly in both directions on an ABI 3730XL automated sequencer (Applied Biosystems, Foster City, CA, USA). Direct sequencing of PCR products generates heterozygous base-calling fluorescence (double peak) chromatograms, which can reveal paralogous genes within individuals (Chang et al. 2012). In this study, none of the ITS sequences obtained from any of the individuals exhibited any double peaks for any of the sites in the chromatograms obtained by direct sequencing.

### DNA sequence alignment and genetic analyses

Nucleotide sequences were aligned using MAFFT version 7 (Katoh et al. 2019) and later adjusted visually. The best-fit models of nucleotide substitution for the alignments were estimated with jModeltest 2.1.10 (Darriba et al. 2012). Maximum-likelihood (ML) analyses and Bayesian inference (BI) algorithms were performed to infer the relationships among the studied *Angelica* taxa. ML analyses were performed using PhyML v. 3.67 (Guindon et al. 2010) for cp and nrDNA haplotypes, and bootstrap consensus values were calculated using 1000 replicates. The BI tree was generated with the program MrBayes 3.2.7 (Huelsenbeck and Ronquist 2001). Two independent Markov chain Monte Carlo (MCMC) runs with 5 × 10^6^ generations were performed for the analysis. Trees were sampled every 1000 generations. The first 25% of the sampled trees were discarded as burn-in, and the remaining trees were used to build a 50% majority-rule consensus tree. FigTree 1.4.4 (Rambaut 2018) and the ggtree package (Yu 2020) in R (http://www.r-project.org) were applied to depict the ML and BI trees. Sequences of *Angelica* from the National Center for Biotechnology Information (NCBI, https://www.ncbi.nlm.nih.gov/; Additional file 1) were used to infer the phylogeny of *Angelica* in Taiwan. Levels of genetic diversity within populations and species were quantified with measures of nucleotide diversity (π) (Nei 1987), haplotype number, and haplotype diversity (Hd) using DnaSP 6 (Rozas et al. 2017). Historical demographic scenarios were analyzed using Tajima’s D with the aid of DnaSP 6.

## Results

### Morphology

**Fig. 2:**
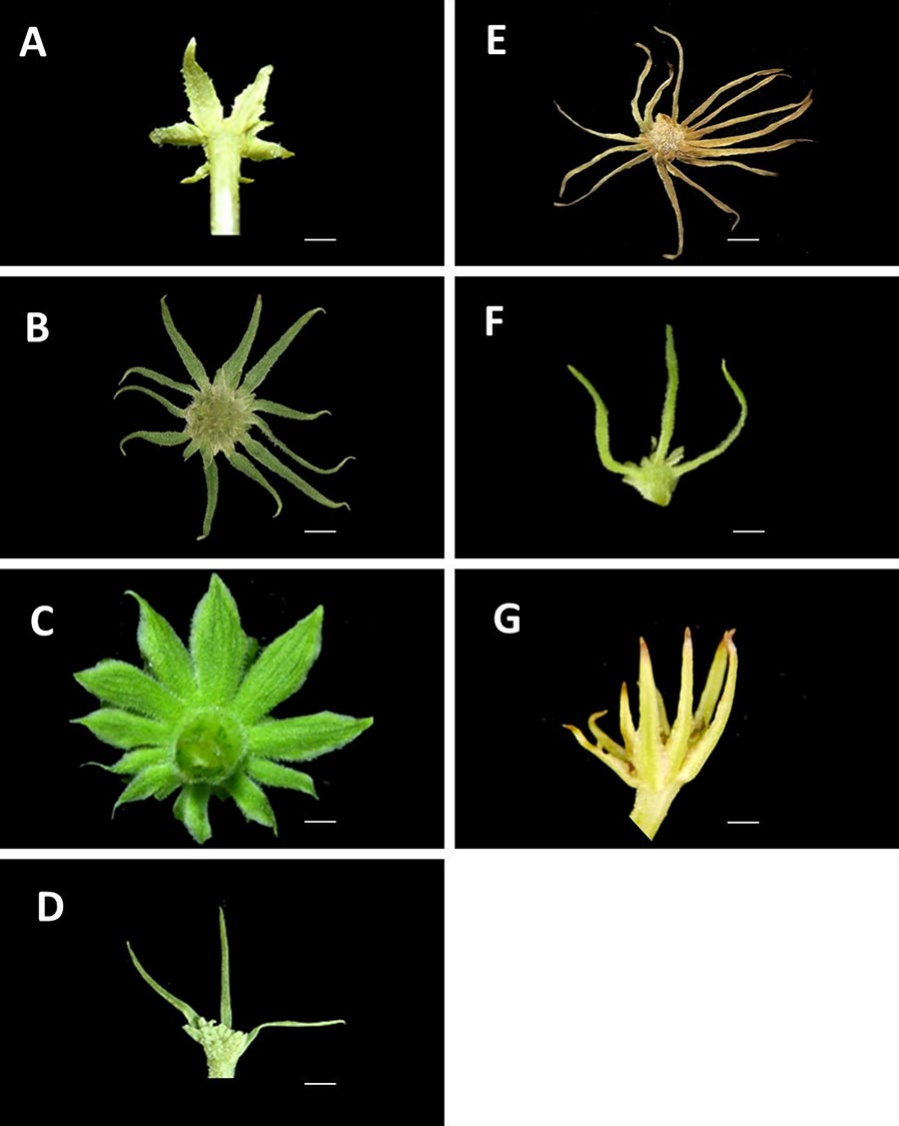
Bracteoles of *Angelica* in Taiwan. Scale bar = 2 mm. **A** *A. aliensis*; **B** *A. dahurica* var. *formosana*; **C** *A. hirsutiflora*; **D** *A. morii*; **E** *A. morrisonicola*; **F** *A. pubescens*; **G** *A. tarokoensis*

**Fig. 3:**
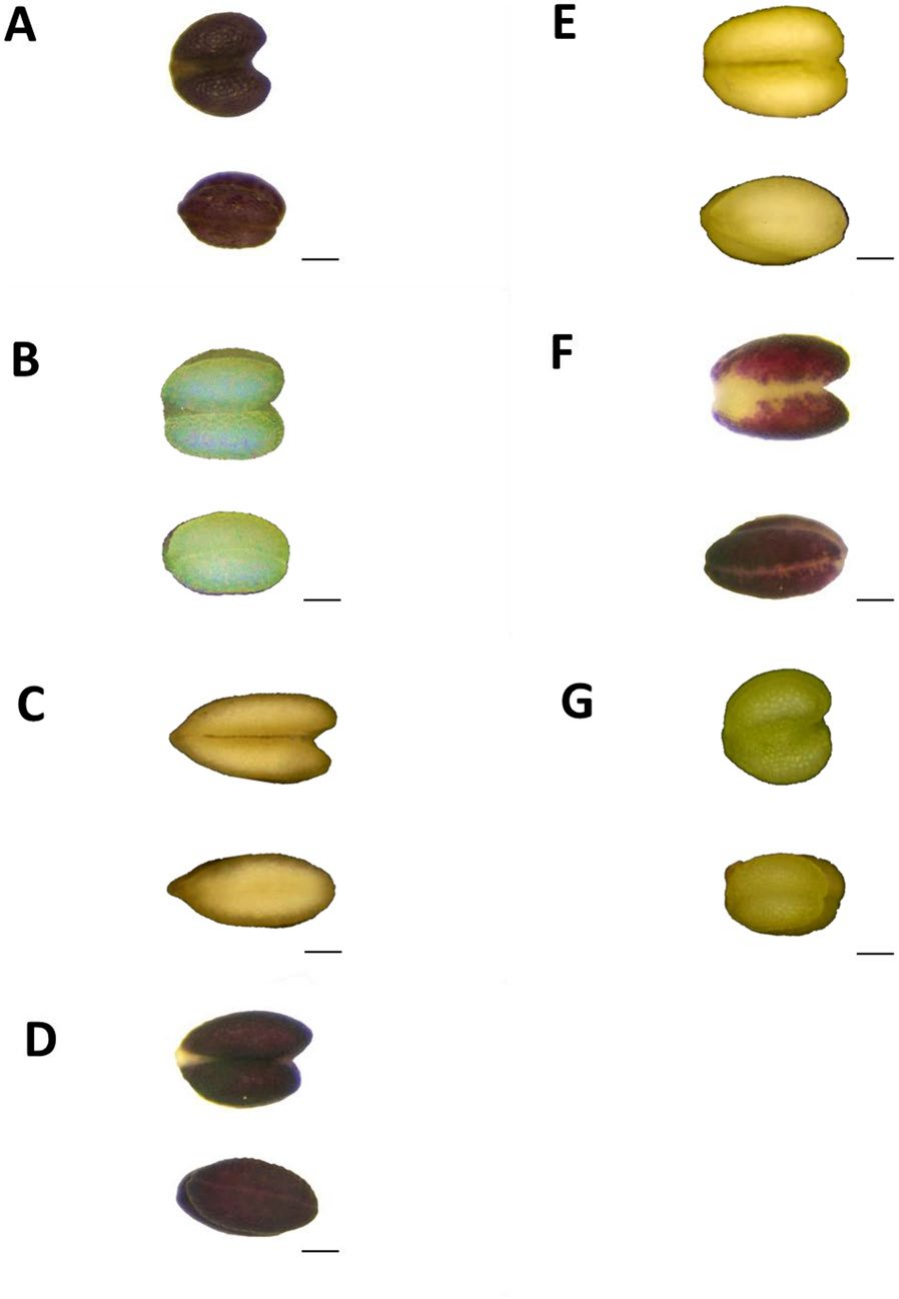
Anthers of *Angelica* in Taiwan. Scale bar = 0.2 mm. **A** *A. aliensis*; **B** *A. dahurica* var. *formosana*; **C** *A. hirsutiflora*; **D** *A. morii*; **E** *A. morrisonicola*; **F** *A. pubescens*; **G** *A. tarokoensis*

The classification of *Angelica* species is controversial due to the presence of several morphological characteristics that exhibit variations within and between species. Plausible morphological features, including leaf morphology, inflorescence structure, stem characteristics, and habitat, contributed to the difficulty of *Angelica* classification. In this study, trichome type on leaf and stem, type of pinnae, reticulate veinlet, bracteole number (Fig. 2) and color of anther (Fig. 3) were applied to distinguish different *Angelica* species in Taiwan (Table 1). The leaf and stem types are hispidulous, glabrous or pubescent. The trichome types are hispid, velutinous, or glabrous. Bracteole types are narrowly triangular, elliptic or linear. The bracteole numbers are 0–4, 6 or 10. Reticulate veinlets are prominent or sunken. Cartilaginous margins of leaflets and ternate pinnae decurrent are absent or present. The anther colors are purple, yellow, or purple/yellow.

A key for *Angelica* in Taiwan is provided (see discussions “Taxonomic treatment of *Angelica* in Taiwan” section below). According to these morphological characteristics, we suggest that *Angelica* in Taiwan can be divided into seven taxa, including *A. aliensis*, *A. dahurica* var. *formosana*, *A. hirsutiflora*, *A. morii*, *A. morrisonicola*, *A. pubescens*, and *A. tarokoensis*.

### Genetic variability of cpDNA and nrDNA

In total, 114 *Angelica* samples from Taiwan were processed. The levels of cp- (*rps16-trnK*) and nrDNA (ITS) genetic diversity were examined*,* as shown in Table 2. The cp- and nrDNA consensus sequences had lengths of 896 bp and 609 bp, respectively. A total of 42 parsimony informative sites were detected in cpDNA, while 105 parsimony informative sites were found in nrDNA. In terms of haplotype diversity, 10 haplotypes were detected in cpDNA (GenBank accession numbers: OR240201–OR240210), and 8 haplotypes of nrDNA (GenBank accession numbers: OR242586–OR242591, OR242594–OR242595) were found*.* Among the different *Angelica* species, *A. aliensis*, *A. dahurica* var. *formosana*, *A. hirsutiflora* and *A. morrisonicola* exhibited single cp- and nrDNA haplotypes. *Angelica morii* and *A. pubescens* had one cp haplotype and two nr haplotypes. *Angelica tarokoensis* had two haplotypes in both cp- and nrDNA. Detailed information can be found in Table 2 and Additional file 1.

In total, the nucleotide (π) and haplotype diversities (Hd) of cpDNA from Taiwan *Angelica* ranged from 0 to 0.00193 and from 0 to 0.526, respectively. *Angelica morii, A. pubescens*, and *A. tarokoensis* had higher π and Hd values than other *Angelica* species in Taiwan. The nucleotide and haplotype diversities of the Taiwan *Angelica* nrDNA ranged from 0 to 0.00063 and from 0 to 0.400, respectively. *Angelica tarokoensis* had higher π and Hd values than other *Angelica* species.

Furthermore, three TNM specimens of *A. dahurica* from China and *Peucedanum japonicum* were processed. Three nrDNA haplotypes (GenBank accession numbers: OR242592, OR242593, and OR242596) were identified in *A. dahurica*, whereas the amplification of cpDNA was unsuccessful. *Peucedanum japonicum* exhibited single cpDNA (OR240211) and nrDNA (OR242597) haplotypes. Detailed information can be found in Additional file 1.

### Phylogenetic analyses

**Fig. 4:**
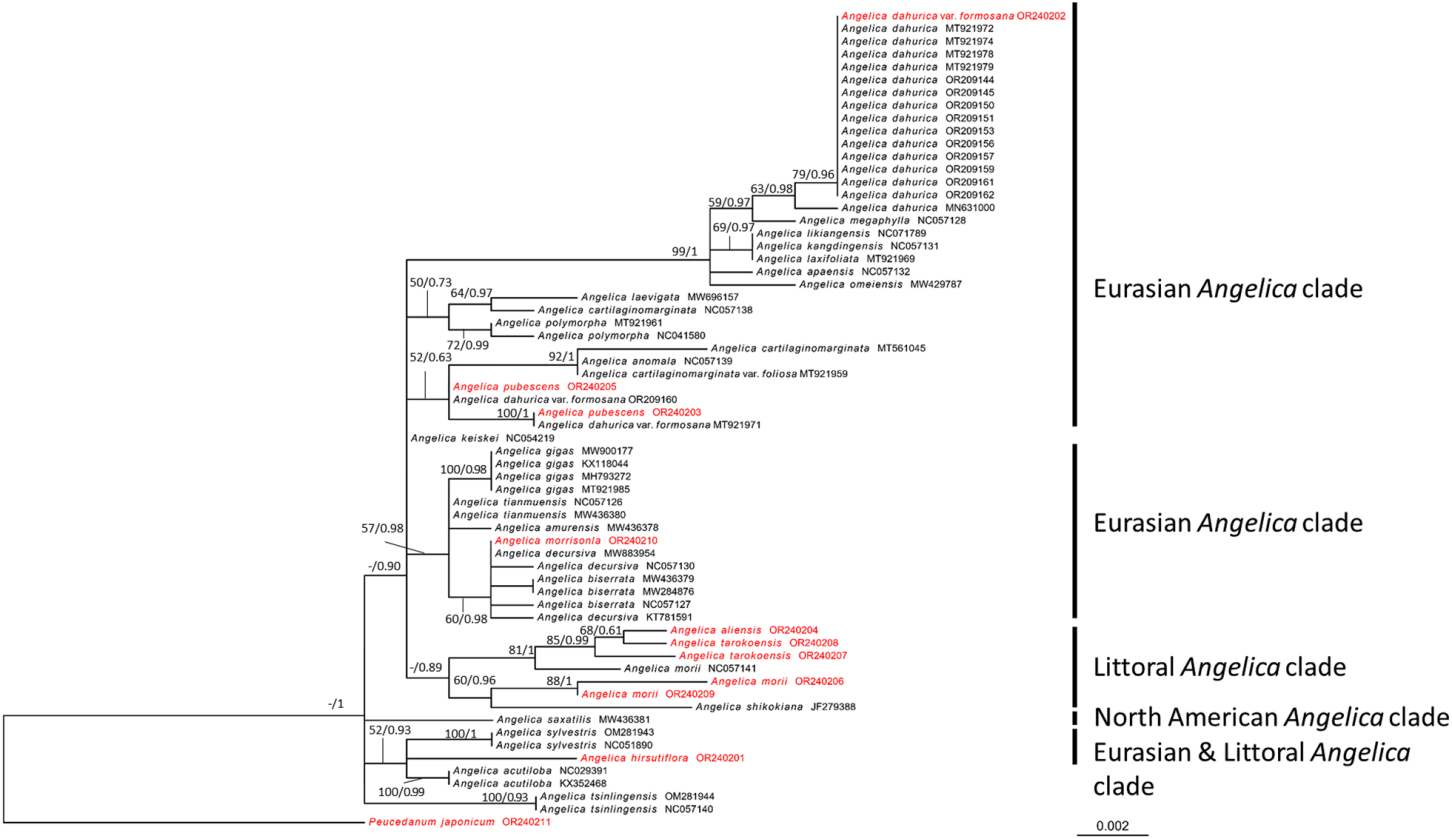
Phylogenetic tree of cpDNA based on a maximum-likelihood analysis. The numbers on the branches are bootstrap values (> 50)/posterior probabilities (> 0.70). Sequences obtained in this study are shown in red, and those retrieved from NCBI are presented in regular black text

**Fig. 5:**
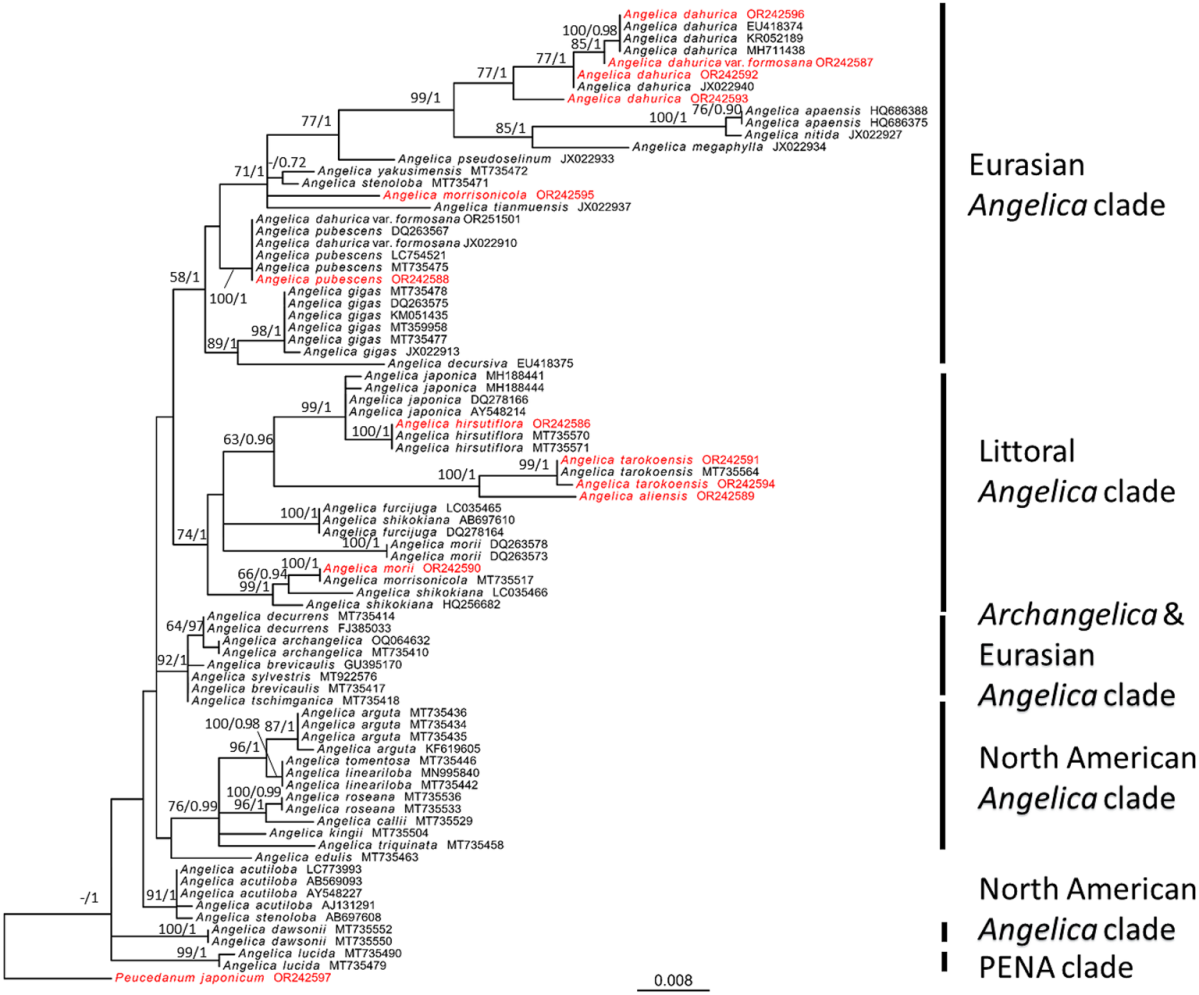
Phylogenetic tree of nrDNA based on a maximum-likelihood analysis. The numbers on the branches are bootstrap values (> 50)/posterior probabilities (> 0.70). Sequences obtained in this study are shown in red, and those retrieved from NCBI are presented in regular black text

A total of 64 cpDNA sequences encompassing 25 taxa were examined. This set of cpDNA sequences comprised 11 sequences generated in this study and an additional 53 sequences sourced from NCBI (Additional file 1). The 81 nrDNA sequences, representing 37 taxa, were analyzed. This set of nrDNA sequences included 9 and 3 TNM sequences from this study, while an additional 69 sequences were obtained from NCBI. The cpDNA (Fig. 4) and nrDNA (Fig. 5) phylogenetic trees of *Angelica* were generated based on ML and BI analyses, with *Peucedanum japonicum* serving as the root. ML trees are presented, as BI trees exhibited consistent topologies with ML trees. The GTR + G mode was determined to be the most suitable model by jModeltest and was used to construct the phylogenetic tree of cpDNA, while the SYM + I + G model was determined to be the most suitable model to construct the phylogenetic tree of nrDNA. The alignments and tree files are provided in Additional files 2 and 3.

The ML tree based on cpDNA revealed the presence of a monophyletic clade of *Angelica. Angelica aliensis* (OR240204) and *A tarokoensis* (OR240207, OR240208) formed a distinct clade closely related to NC057141 (*A. morii* from NCBI). *Angelica dahurica* var. *formosana* (OR240202) was grouped with MN631000, MT921972, MT921974, MT921978, MT921979, OR209144, OR209145, OR209150, OR209151, OR209153, OR209156, OR209157, OR209159, OR209161, and OR209162 (*A. dahurica* from NCBI). *Angelica hirsutiflora* (OR240201) appeared to be distantly related to the other clades. *Angelica morii* (OR240206, OR240209) formed a monophyletic clade that was more closely related to *A. shikokiana* (JF279388) than to NC057141 (*A. morii* from NCBI). *Angelica morrisonicola* (OR240210) was grouped with NC057127, MW284876, and MW436379 (*Angelica biserrata*) and NC057130, MW883954, and KT781591 (*Angelica decursiva*). *Angelica pubescens* (OR240203 and OR240205) were grouped with NC057139 (*Angelica anomala*), MT921971 and OR209160 (*A. dahurica* var. *formosana*), MT561045 (*Angelica cartilaginomarginata*) and MT921959 (*Angelica cartilaginomarginata* var. *foliosa*).

The phylogenetic tree constructed based on nrDNA. *Angelica aliensis* (OR242589) was closely related to *A. tarokoensis* (OR242591, OR242594), which formed a monophyletic group with MT735564 (*A. tarokoensis* from NCBI). *Angelica dahurica* var. *formosana* (OR242587), and *A. dahurica* (OR242592, OR242593, and OR242596) formed a monophyletic group with EU418374, MH711438, and KR052189 (*A. dahurica* from NCBI). *Angelica hirsutiflora* (OR242586) formed a monophyletic group with MT735570 and MT735571 (*A. hirsutiflora* from NCBI) and was found to be related to AY548214, DQ278166, MH188441 and MH188444 (*A. japonica* from NCBI). *Angelica morii* (OR242590), which was not closely related to DQ263573 and DQ263578 (*A. morii* from NCBI), was grouped with MT735517 (*A. morrisonicola* from NCBI). *Angelica morrisonicola* (OR242595) did not form a monophyletic group with MT735517 (*A. morrisonicola* from NCBI). *Angelica pubescens* (OR242588) was related to DQ263567, MT735475 and LC754521 (*A. pubescens* from NCBI) and OR251501 and JX022910 (*A. dahurica* var. *formosana* from NCBI).

Interestingly, the nrDNA phylogenetic tree revealed that *Angelica* in Taiwan can be divided into two major clades (the Eurasian *Angelica* and littoral *Angelica* clades). *Angelica dahurica* var. *formosana*, *A. morrisonicola*, and *A. pubescens* were clustered with the Eurasian *Angelica* clade, suggesting their closer relationship to other *Angelica* species from Eurasia. Conversely, *A. aliensis*, *A. hirsutiflora*, *A. morii*, and *A. tarokoensis* clustered with the littoral *Angelica* clade, indicating their affiliation with *Angelica* species found in coastal regions.

## Discussion

### Taxonomic revision of *Angelica* in Taiwan

*Angelica dahurica* var. *formosana*, which is endemic to the northern part of Taiwan, had been divided into different taxonomic states, e.g., *A. formosana*, *A. dahurica, A. pubescens* var. *glabra*, revealing its undistinguished morphology (Liu and Kao 1977; Kao 1993). It is also considered an endangered species (category of vulnerable, VU) (Editorial Committee of the Red List of Taiwan Plants 2017). In this study, three putative populations (DT, LK and NZ) of *A. dahurica* var. *formosana* were sampled to revise the species delimitation. The main population (DT) is located in the district of Yangmingshan National Park with proper protection, while LK and NZ are located in unprotected regions. The cpDNA and nrDNA haplotypes observed in the DT population (cpDNA: OR240203; nrDNA: OR242588) were not found in the other two populations (cpDNA: OR240202; nrDNA: OR242587). This lack of shared haplotypes suggested genetic differentiation between the DT population and the LK and NZ populations. The cpDNA and nrDNA ML trees revealed that DT is clustered with DY and FS (cpDNA: OR240205, nrDNA: OR242588), which is considered *A. pubescens* (Chen 2008), while LK and NZ are clustered with *A. dahurica*. These findings implied possible misidentification between *A*. *dahurica* var. *formosana* and *A. pubescens*. The morphological revision of wild populations and herbarium specimens revealed differences between DT and the other two populations. Morphological characters such as velutinous stems and yellow to purple colors of the anthers of DT and *A. pubescens* (DY and FS) can be distinguished from LK and NZ, with their hispid stems and anthers that are only yellow in color. The morphological and molecular evidence suggested that DT should be treated as *A. pubescens*, while LK and NZ should be treated as *A. dahurica* var. *formosana*. These findings are noteworthy, as *A. pubescens* is typically considered to be distributed in the central mountains of Taiwan.

Both cpDNA and nrDNA sequences of *A. dahurica* var. *formosana* from NCBI (cpDNA: MT921971 and OR209160, nrDNA: JX022910 and OR251501) were grouped with *A. pubescens.* The MT921971 and JX022910 sequences were extracted from specimen HLQA10042 (CMMI), which was located in Hsinchu City, Taiwan. The location of this sample could be a typo, as Hsinchu City is urbanized. The OR209160 sequence was collected in Nantou, Taiwan, while information for OR251501 was not available. It is possible that *A. dahurica* var. *formosana* from NCBI was misidentified, as *A. pubescens* was never formally described in the flora of Taiwan (Kao 1993). Therefore, these sequences of *A. dahurica* var. *formosana* from NCBI should be treated as *A. pubescens*.

*Angelica morrisonicola* is an endemic species that specifically inhabits elevations ranging between 3000 and 3500 m. In contrast, *A. morrisonicola* var. *nanhutashanensis* has a more limited distribution, being found only in the Nahutashan area (NH) at approximately 3700 m above sea level. It is morphologically distinguished from *A. morrisonicola* due to its small leaves densely covered in hispid on both surfaces (Liu et al. 1961; Kao 1993). In this study, populations of YS and NH exhibited identical cpDNA (OR240210) and nrDNA (OR242595) haplotypes, indicating that they cannot be differentiated at the molecular level (Table 2). Chen (2008) proposed that the densely hispid leaves of *A. morrisonicola* var. *nanhutashanensis* showed variability in different microhabitats and were not stable characteristics for distinguishing it from *A. morrisonicola.* Taking into account both the morphological and molecular evidence, it has been suggested that *A. morrisonicola* and *A. morrisonicola* var. *nanhutashanensis* should be considered a single taxon, namely, *A. morrisonicola*.

*Angelica morii* is an endemic species found in the central mountains of Taiwan at elevations ranging from 2500–3000 m. Liao et al. (2022) placed both *A. morii* and *A. morrisonicola* within the littoral *Angelica* clade. The exclusive haplotypes of *A. morii* (cpDNA: OR240206, OR240209; nrDNA: OR242590) and *A. morrisonicola* (cpDNA: OR240210; nrDNA: OR242595) in this study were compared with those in a previous study (Liao et al. 2022), showing different groupings. In the cpDNA tree, *A. morii* from Taiwan was more closely related to *A. shikokiana* (JF279388) than to *A. morii* from Fujian (NC057141). *Angelica morrisonicola* from Taiwan was not grouped with the littoral *Angelica* clade. In the nrDNA tree, *A. morii* (OR242590) was found to be closely related to MT735517, representing *A. morrisonicola*, rather than to DG263573 and DG263578, which represent *A. morii* from Fujian. Conversely, *A. morrisonicola* (OR242595) displayed a closer association with the Eurasian *Angelica* clade than being grouped within the littoral *Angelica* clade, as proposed by Liao et al. (2022). Species descending from a common ancestor are expected to differentiate from each other and eventually achieve reciprocal monophyly (Ting et al. 2000; Huang et al. 2011). The paraphyletic grouping observed for MT735517 could be attributed to the misidentification of herbarium samples (Wang 2018; de Almeida et al. 2023). The MT735517 sequence was extracted from Z.H. Li et al. 14080302 (SZ) without any detailed information. Therefore, it is suggested that the MT735517 sample be reidentified, which would likely place it within *A. morii*. The distinct grouping of *A. morii* from Taiwan, separate from *A. morii* from Fujian (cpDNA: NC057141; nrDNA: DG263573 and DG263578), raises the possibility of taxonomic issues that could benefit from further morphological revision. Additionally, the close relationships observed between *A. morrisonicola* and *A. dahurica*, as well as *A. dahurica* var. *formosana*, suggest the need to regroup them within the Eurasian *Angelica* clade.

*Angelica tarokoensis* is an endemic species found in the eastern and southern mountains of Taiwan at elevations ranging from 400–2000 m. It is also considered an endangered species (category of endangered, EN) (Editorial Committee of the Red List of Taiwan Plants 2017). *Angelica aliensis*, proposed by Chen (2008), is a new taxon separated from *A. tarokoensis* and is restricted to Wutai, the southern mountains of Taiwan at elevations ranging from 800 to 1200 m. *Angelica aliensis,* with 2–3 pinnately compound leaves and purple-colored anthers, can be separated from *A. tarokoensis* with 1–2 pinnately compound leaves and yellow-colored anthers. In the nrDNA ML tree, *A. tarokoensis* formed a monophyletic clade and showed a close relationship with *A. aliensis.* The results obtained from the analysis of the morphological and molecular characteristics supported the notion that *A. aliensis* and *A. tarokoensis* are closely related but represent distinct taxa. Their geographic isolation may be attributed to the divergence of A. *aliensis* and *A. tarokoensis.* Additionally, *A. aliensis* morphologically resembles *A. shikokiana* Makino ex Y. Yabe, which is distributed in Japan (Hiroe and Constance 1958). The morphological and molecular evidence indicated that *A. aliensis* and *A. shikokiana* (cpDNA: JF279388; nrDNA: AB697610–AB697611) are distinct taxa.

*Angelica hirsutiflora* is an endemic species found in the coastal areas of the northern part of Taiwan. It is also considered an endangered species (VU) (Editorial Committee of the Red List of Taiwan Plants 2017). Despite being geographically isolated by sea (over 200 km), it shows significant genetic homogeneity, with only a single haplotype observed in cpDNA (OR240201) and nrDNA (OR242586). Interestingly, the nrDNA ML trees also revealed a striking affinity with another species called *A. japonica*, findings that are consistent with previous research by Seo et al. (2005). Based on these results, it is hypothesized that *A. hirsutiflora* and *A. japonica* likely share a common ancestor, making them the most recent relatives among coastal *Angelica* species in the region. These discoveries add to our understanding of the evolutionary history and genetic relationships of these fascinating coastal *Angelica* species in Taiwan.

A total of seven taxa in Taiwan were confirmed, and their taxonomic states were investigated. Among these taxa, three were placed within the Eurasian *Angelica* clade, namely, *A. dahurica* var. *formosana, A. morrisonicola*, and *A. pubescens*. The remaining four taxa were categorized into the littoral *Angelica* clade, which included *A. aliensis, A. hirsutiflora, A. morii* and *A. tarokoensis*. Notably, the analysis revealed that *A. morrisonicola* showed a close relationship with *A. dahurica* var. *formosana*, rather than with the other members of the littoral *Angelica* clade. These two taxa also exhibited a significant affinity with *A. pubescens*, indicating that *A. morrisonicola* should be assigned to the Eurasian *Angelica* clade. Additionally, the study identified *A. aliensis* as closely related to *A. tarokoensis*, while *A. hirsutiflora* showed a close relationship with *A. japonica*. Finally, among the littoral *Angelica* clade in Taiwan, *A. morii* was found to be the most distantly related species. These findings provide valuable insights into the evolutionary relationships and groupings of *Angelica* species in Taiwan, shedding light on their phylogenetic affinities and distributions within distinct clades.

### Comparison between cpDNA and nrDNA trees

In this study, the species delimitations of *Angelica* in Taiwan were revised in accordance with the monophyletic groups formed. For example, A. *morii* formed a monophyletic group and was closely related to *A. shikokiana* in both the cpDNA and nrDNA trees. In terms of nrDNA, all *Angelica* in Taiwan had their own haplotypes and could be distinguished from other *Angelica* species. However, *A. aliensis*, *A. pubescens* and *A. tarokokenis* did not form monophyletic groups in cpDNA. Furthermore, the phylogenetic relationships of *Angelica* were not identical between the cpDNA and nrDNA trees. Three possible reasons could contribute to this phylogenetic inconsistency. First, there was a discrepancy in taxa number between cpDNA and nrDNA. Specifically, we compared 25 taxa from cpDNA and 37 taxa from nrDNA. Certain DNA information for *Angelica* taxa was not present in the NCBI database. For example, *A. hirsutiflora* was closely related to *A. japonica* in the nrDNA tree, although the cpDNA data for *A. japonica* were absent in the NCBI. The potential misidentification of taxa could have played a role in the observed inconsistency (Lal and Lal 2011; Stavrou et al. 2018). Some possible misidentifications were proposed in this study (see the discussion in the “Taxonomic revision of Angelica in Taiwan” section above). Second, incomplete lineage sorting may have contributed to the inconsistency. The evolutionary rates of chloroplast and nuclear genes differ (Huang et al. 2012). Concerted evolution of nuclear ITS, which homogenizes the sequence (Nei et al. 2000; Naidoo et al. 2013), may lead to species delimitations concordant with those based on morphology (Okuyama et al. 2005). In this study, *A. pubescens* was grouped with *A. anomala* and *A. cartiaginomarginate* in cpDNA but formed a monophyletic group in nrDNA. Incomplete lineage sorting of cpDNA may be attributed to the inconsistency of *A. pubescens*. Third, chloroplast capture via hybridization is attributed to changes in the chloroplast genome and affects the reconstruction of cpDNA phylogeny (Soltis and Kuzoff 1995; Padgett et al. 1999). Liao et al. (2013, 2022) proposed that chloroplast capture may be a common reason for the inconsistencies between cpDNA and nrDNA phylogenetic trees of *Angelica*. In this study, *A. aliensis* and *A. tarokoensis*, which were closely related in nrDNA, were found to be mixed in cpDNA. These two taxa may have overlapping geographic distributions, despite their rarity. Chloroplast capture may be the main reason for this phenomenon. Therefore, the nrDNA tree displayed a closer congruence with the morphological data and thus was considered to be a more reliable representation of the evolutionary relationships in this study.

### Genetic diversity of *Angelica* in Taiwan

Taiwan, as a glacial refuge, provides shelters for many species and harbors substantial species diversity (Chiang and Schaal 2006). Numerous studies have highlighted the north-central mountainous region of Taiwan as a significant glacial refuge characterized by higher levels of genetic diversity in various species. Among these species are *Abies kawakamii* (Shih et al. 2007), *Chamaecyparis formosensis* (Huang et al. 2022a), *Cunninghamia konishii* (Chung et al. 2004), *Euphrasia nankotaizanensis* (Chen et al. 2023), *Machilus thunbergii* (Wu et al. 2006), *Michelia formosana* (Lin 2001), *Rhododendron pseudochrysanthum* (Huang et al. 2011) and *Trochodendron aralioides* (Huang et al. 2004). These findings underscore the importance of this area in providing a refuge for diverse plant species during periods of glacial cycles.

Several molecular markers, such as nuclear DNA and plastid fragments, have been widely applied in the molecular identification, systematics and phylogeny of *Angelica* (Feng et al. 2009; Chen et al. 2010, 2017; Liao et al. 2012, 2013, 2022; Jiang et al. 2022). Few studies have focused on population genetic structure. Minami et al. (2023) proposed that only three chloroplast *atpF-atpA* haplotypes were identified among 106 specimens of *A. acutiloba* and *A. acutiloba var. iwatensis*. Moreover, DNA fingerprinting technology has been applied in the evaluation of the population genetics of *Angelica* (Mei et al. 2015; Liu et al. 2020). Huang et al.(2022b) proposed that *A. dahurica* in the wild possesses moderate genetic diversity (observed heterozygosity: 0.523), higher than that of cultivars (0.348–0.397). Liu et al. (2020) proposed that a high level of genetic diversity occurred in a wild *A. biserrata* germplasm. This study revealed the low genetic diversity of *Angelica* in Taiwan, as evidenced by both the cpDNA and nrDNA markers (Table 2). Specifically, *A. hirsutiflora* and *A. dahurica* var. *formosana*, which primarily inhabit coastal and low-altitude areas, exhibited no detectable genetic diversity. Notably, a population of *A. dahurica* var. *formosana* previously recorded in Taipei may have experienced local extinction due to human disturbances and habitat degradation. However, the remaining populations of *A. dahurica* var. *formosana* can still be found in less-disturbed areas, such as LK and NZ. Furthermore, we found positive values of Tajima’s D in the cpDNA of *A. morii* and *A. pubescens*, indicating significant deviations from population equilibrium. This result suggested the possibility of population subdivision or recent population bottlenecks (Tajima 1989; Fay and Wu 1999). Genetic drift, such as bottlenecks, may be a contributing factor leading to the fixation or reduced levels of genetic diversity of *Angelica* populations in Taiwan.

It is often observed that species with a wide distribution may harbor higher genetic diversity (Soltis and Soltis 1991; Wang 2020). *Angelica hirsutiflora*, despite having a broad geographic range of over 200 km, displayed no genetic variation, while *A. morii*, *A. pubescens* and *A. tarokoensis*, distributed in medium- to high-altitude areas, displayed higher cpDNA genetic diversity. One possible explanation for these findings is habitat differentiation. Zhang and He (2013) utilized three chloroplast DNA fragments to infer the phylogeography of *A. nitida* endemic to the Qinghai‒Tibet Plateau. The complex topography and spatial isolation hindered the gene flow of *A. nitida*, which was attributed to the high degree of genetic differentiation. The diverse environments found in mountainous regions provide varied habitats for these *Angelica* taxa, leading to differences in their genetic diversity. For instance, *A. pubescens* displayed different haplotypes in different regions, with DT representing the northern and low-elevation populations, while DY and FS from the central mountain range of Taiwan preserved different haplotypes. These results align with previous studies (Chiang et al. 2006; Huang et al. 2011; Chen et al. 2023), which emphasized the central mountain range of Taiwan as a significant reservoir of plant biodiversity. Overall, the study provides valuable insights into the relationships between geographic distribution, habitat differentiation, and genetic diversity in *Angelica* populations, contributing to our understanding of the factors influencing species diversification and distribution patterns in Taiwan.

### Conservation strategies of *Angelica* in Taiwan

The goal of conservation is to ensure the preservation of biodiversity (Avise 1989). A higher level of genetic diversity helps maintain the continuation of species and populations. Prioritizing the assessment of population differentiation is essential for gaining insights into genetic diversity. Therefore, information about the genetic structures of threatened species helps managers formulate appropriate conservation and management strategies (Milligan et al. 1994; Wang 2020). Identification of conservation units is essential in formulating conservation strategies (Funk et al. 2012; Coates et al. 2018). Our results suggested that a low level of genetic diversity was detected in *Angelica* in Taiwan. *Angelica dahurica* var. *formosana*, *A. hirsutiflora* and *A. tarokoensis* were categorized as threatened species (Editorial Committee of the Red List of Taiwan Plants 2017). Various conservation strategies for *Angelica* in Taiwan have been proposed based on genetic structure, distribution and the level of disturbance.

The morphology, distribution and genetic structure of *A. dahurica* var. *formosana* were revised to distinguish it from *A. pubescens.* It is suggested that *A. dahurica* var. *formosana,* with its identical nuclear and chloroplast haplotypes (Table 2), should be treated as a single conservation management unit. Furthermore, the LK population, which has a larger population size and has been subjected to less human disturbance, should be given higher priority for further conservation, while the NZ population is experiencing greater human disturbance. Measures such as habitat protection, germplasm collection and controlling overgrazing should help maintain the population size of *A. dahurica* var. *formosana.*

*Angelica hirsutiflora* is restrictedly distributed in coastal areas in northern Taiwan and outlying islands. Due to the presence of identical nuclear and chloroplast haplotypes among sampled populations (Table 2), it is suggested that *A. hirsutiflora* should be treated as a single conservation management unit. Habitat protection and germplasm collection measures should help maintain the population size of *A. hirsutiflora.*

It is also suggested that *Angelica tarokoensis*, with its different nuclear chloroplast haplotypes (Table 2), should be treated as two conservation management units corresponding to the native distribution areas. Inventory, germplasm collection and habitat protection measures should help maintain the population size of *A. tarokoensis.*

*Angelica aliensis*, which is restrictedly distributed in southern Taiwan, is a new taxon proposed in this study. Due to its small population size and unique nuclear and chloroplast haplotypes, it is suggested that *A. aliensis* should be categorized as a threatened species on the red list of vascular plants and treated as a single conservation management unit. Inventory, germplasm collection and habitat protection measures should help maintain the population size of *A. aliensis.*

Protected areas contribute to the maintenance of biodiversity worldwide (Liao et al. 2013). The nonthreatened *A. morii, A. morrisonicola* and *A. pubescens,* primarily distributed in mountain areas, are located inside protected areas. However, all threatened *Angelica* in Taiwan, except *A. tarokoensis*, are found outside protected areas. Given the lack of legal protection, priority should be accorded to germplasm collection for endangered *Angelica* species. Wang (2020) proposed three management units of *Paeonia decomposita* and provided recommendations for germplasm collection. Liu et al. (2020) constructed a core collection of *A. biserrata* using SSR and metabolic data. Therefore, further genetic and metabolic studies of *Angelica* are necessary for germplasm collection.

### Taxonomic treatment of *Angelica* in Taiwan

Based on our morphological and molecular studies, the genus *Angelica* of Taiwan is taxonomically revised. We provide key to taxa, synopsis, phenology, and distribution for each taxon of Taiwan. Description and examined specimens of the new species, *A. aliensis*, and *A. pubescens*, a taxon which was never described in the flora of Taiwan are also giving.

1a. Leaves with trichomes along the midribs and lateral veins; stems and petioles pubescent …………………………………………2

1b. Leaves, stems and petioles glabrous ……………………………………………………………………………………………………………………………………………………………………………………5

2a. Stems and leaves hispid ……………………………………………………………………………………………………………………………………………………………………………………………………………………3

2b. Stems and leaves velutinous …………………………………………………………………………………………………………………………………………………………………………………………………………4

3a. Pinnae widely ovate to obovate; bracteoles elliptic; petals dorsally hirsute; maritime plant ……………………………………………………………………………………………………………………………………………………………………………………*A. hirsutiflora*

3b. Pinnae elliptic to rhombic; bracteoles linear; ternate pinnae slightly decurrent at base ……………………………………………………………………………………………………………………………………………………………………………………*A. dahurica* var.* formosana*

4a. Bracteoles about 10; anther yellow …………………………………………………………………………………………………*A. morrisonicola*

4b. Bracteoles 0–4; anther purple mingle with yellow ………………………………………………………………………*A. pubescens*

5a. Reticulate veinlets sunken except midrib; anther purple ………………………………………………………………*A. morii*

5b. Reticulate veinlets prominent 6

6a. Anthers purple; leaflets without cartilaginous margins …………………………………………………………*A. aliensis*

6b. Anthers yellow; leaflets with white colored cartilaginous margins; hispid on veins …………………………………………………………………………………………………………………………………………………………………………………………………………………………………………*A. tarokoensis*

**Fig. 6:**
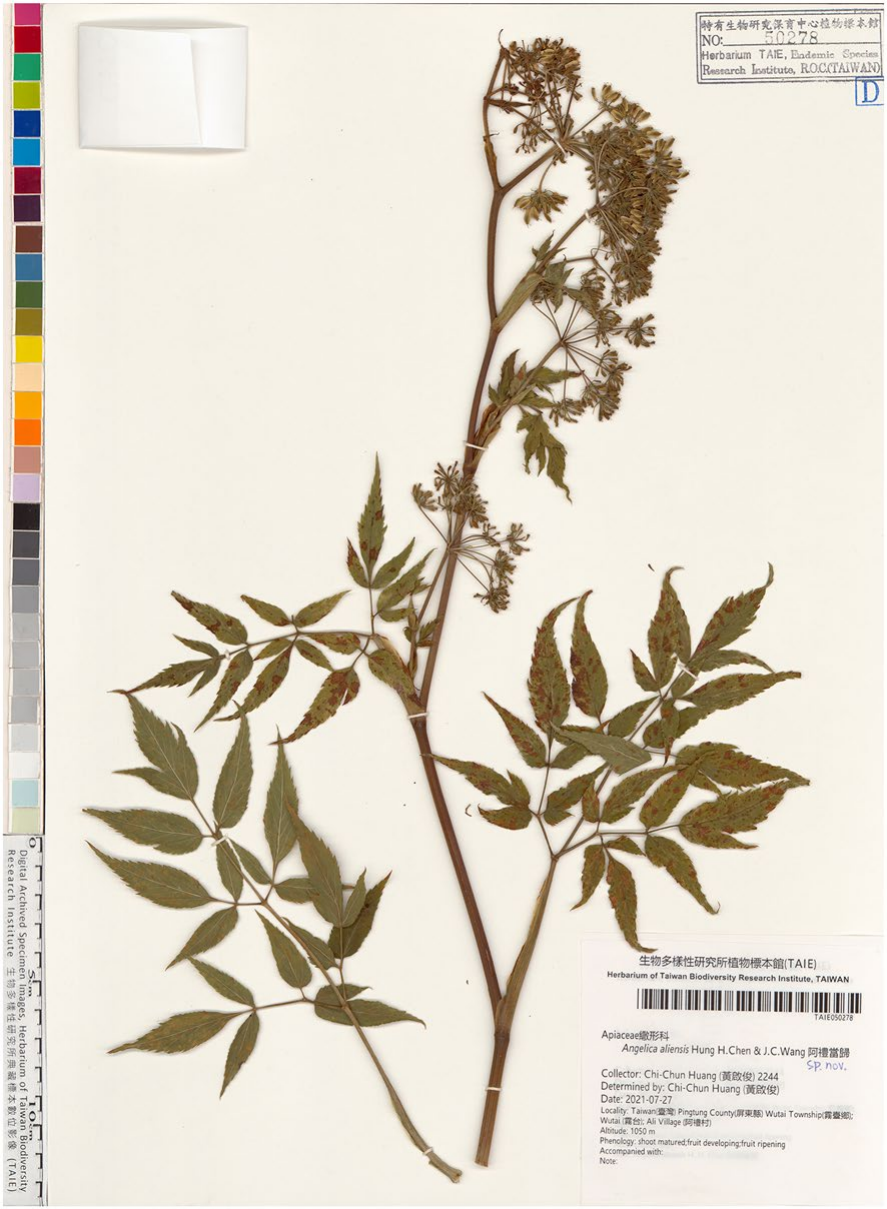
Type specimen of* Angelica aliensis* H. H. Chen & J. C. Wang

**Fig. 7:**
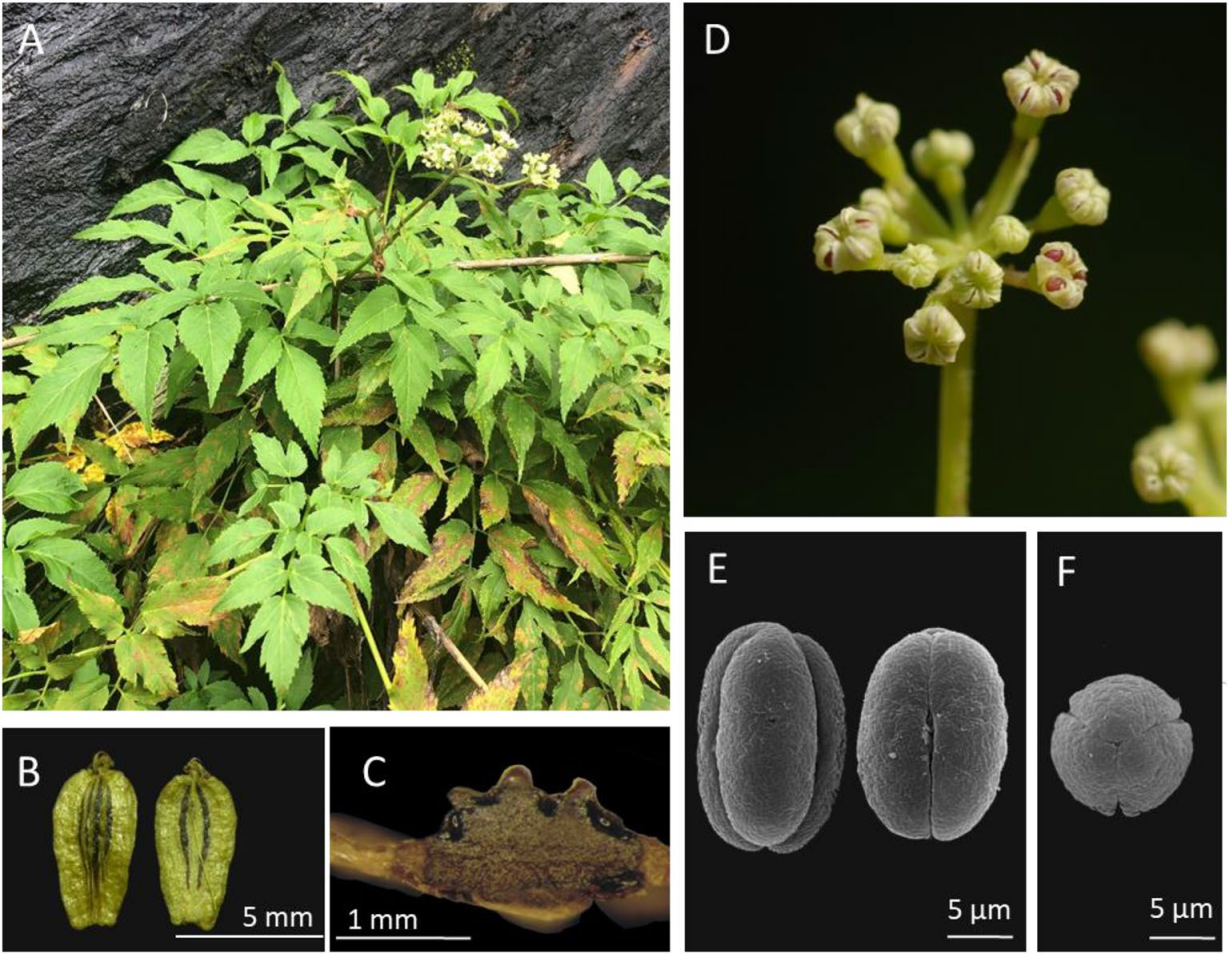
*Angelica aliensis* H. H. Chen & J. C. Wang. **A** habit; **B** schizocarp; **C** cross section of mericarp; **D** flowering inflorescence; **E** SEM photographs of pollen grains (equatorial view); **F** SEM photographs of pollen grains (polar view)

1. ***Angelica aliensis*** H. H. Chen & J. C. Wang, *sp. nov.* (Figures 6 and 7)

-TYPE: Pingtung Hsien: Wutai Hsiang: Ali village, alt. ca. 1000-1100 m, Jul 27, 2021, *C. C. Huang 2244* (Holotype: TAIE)

*Angelica aliensis* resembles *A. shikokiana* Makino ex Y. Yabe, but differs from the latter by having 4–6 narrowly-triangular (vs. 0–2, linear) bracteoles, obovate (vs. oblong to ovate) fruits, and serrate (vs. crenate) leaf margin. *Angelica aliensis* is also similar to *A. tarokoensis* Hayata, but can be easily distinguished by its leaf blade chartaceous (vs. subcoriaceous), leaf 2–3-ternate pinnate (vs. 1–2-ternate pinnate), margin without white-colored fringe, fruit obovate (vs. narrow oblong), anther purple (vs. yellow), and bracteoles 4–6 (vs. 10). We are able to compare them in detail (see Table 3).

**Table 3:** Morphological comparison of *Angelica aliensis, * *A. shikokiana* and* A. tarokoensis*

	*A. aliensis*	*A. shikokiana*	*A. tarokoensis*
Distribution	Taiwan, Pingtung County	Japan, Shikoku & Kyushu	Taiwan, Hualien County
Texture	Chartaceous	Subcoriaceous	Subcoriaceous
Decompound	2–3-ternate-pinnate	1–2-ternate-pinnate	1–2-ternate-pinnate
With white-colored fringe	No	No	Yes
Shape of leaflet	Lanceolate to ovate	Lanceolate to narrowly ovate	Lanceolate
Size of leaflet	3–8 × 2–3	4–8 × 1.5–3	3–7 × 1–2
Long × wide (cm)			
Margin	Serrate	Crenate	Serrate
Anther	Purple	Purple	Yellow
Number of rays	20–30	15–30	10–20(26)
Shape of bracteoles	Narrowly-triangular	Linear	Narrowly-triangular
Number of bracteole	4–6	0–2	ca. 10
Shape of seed	Obovate	Oblong to ovate	Narrowly oblong
Size of seed long × wide (mm)	6–7 × 3–4	5–8 × 3–4	7–8 × 3–4

Herbs perennial. Root tuberous. Stems 30–100 cm high, glabrous. Leaves 30–60 cm long, 2–3-ternate-pinnate, chartaceous, glabrous or with hispid along main veins adaxially; pinnae lanceolate to ovate, 4–8 cm long, 2–3 cm wide, acuminate at apex, obtuse or attenuate at base, the margins mucronate-serrate, the low pinnae usually ternate; reticulate veinlets prominent, petioles glabrous. Umbels compound, rays 20–30, about 5 cm long, subequal; peduncles hispid; bracts 1–2, linear-lanceolate, about 2 cm long; umbellules 2 cm in diameter; pedicels about 10–20; bracteoles 4–6, narrowly-triangular, 1 cm long. Flowers white; calyx-teeth deltoid; petals ovate to obovate, with a narrowly inflexed apex; anther purple; ovary glabrous to hispid. Fruit oblanceolate, glabrous, 6–7 mm long, 3–4 mm wide, lateral ribs broadly thin-winged, narrower than seed, dorsal ribs filiform. Vittae solitary in the interval, 2 in the commissure. Seeds strongly compressed dorsally, inner face plane to concave.

**Phenology**: Flowering in July to August, fruiting in August to September.

**Distribution**: Endemic in Taiwan, so far only found in mountain area of Ali, Pingtung County.

**Specimens examined**: PINGTUNG: en route from Ali to Hsiaokueihu ca. 1 km from Ali, alt. 1200 m, Jun. 26, 2002, *W. C. Leong, 2995* (HAST); Ali to Hsiaokueihu, alt. 1200–1600 m, Aug. 31, 1932, *S. Suzuki, 11,131* (TAI); en route from Ali to Hsiaokueihu ca. 2 km from Ali, alt. 1200–1600 m, Sep. 1, 2007, *H. H. Chen 566* (TNU); same loc., alt. 1200–1600 m, Sep. 1, 2007, *H. H. Chen 567* (TNU); Wutai, alt. 1000–1100 m, Jul 27, 2021, *C. C. Huang 2244* (TAIE).

**Notes:** *Angelica aliensis* was first collected in 1932 by Japanese taxonomist S. Suzuki at the same locality with our collection. He identified it as *A. tarokoensis* probably due to their similarity in having glabrous plant surface, similar leaflet shape and margin and, most possibly, lacking enough specimens to evaluate the variation range of *A. tarokoensis.* Since Suzuki’s collection, more specimens were accumulated in the herbaria of Taiwan. Therefore, we are able to compare them in detail (see Tables 1 and 3) and consequently draw the conclusion that they are different species. Moreover, molecular phylogenetic study reveals that *A. tarokoensis* formed a monophyletic clade and showed a close relationship with *A. aliensis*. Conclusively, both morphological and molecular results supported that *A. aliensis* and *A. tarokoensis* are closely related but distinct species. In addition, *A. tarokoensis* is restricted in eastern Taiwan while *A. aliensis* is confined in southern Taiwan. Geographic isolation may attribute to their divergence.

***2. Angelica dahurica*** (Fisch.) Benth. & Hook. var. ***formosana*** (Boiss.) Yen, Taiwan Pharm. Assoc. 17(2): 68, *f. 1.* 1965; Liu & Kao in H. L. Li et al., Fl. Taiwan 3: 940,*.* 1977. *excl*. *pl. 877*; Shan & Sheh in S. L. Shan et al*.,* Fl. Reipubl. Popularis Sin. 55(3): 35. 1992; Kao in T. C. Huang et al., Fl. Taiwan 2nd ed. 3: 1011, 1993. *excl. pl. 504*; Pan & Watson in Z. H. Wu et al., Fl. China 14: 169. 2005.

*Angelica formosana* Boiss. Bull. Soc. Bot. 56: 354. 1909; Hayata, Icon. Pl. Form. 10: 24. 1912. 1954.-TYPE: Formose, Mazuyama (as Muzuyana), May 1903, *Faurie 117* (as 128) (Syntype: P, 2 sheets, image!); Pashiran, Jun. 1903, *Faurie 117* (Syntype: not traced, probably No. 128)

*Angelica dahurica* auct. non (Fisch.) Benth. & Hook.: Odashima, J. Soc. Trop. Agri. 7: 82. 1935. *pro parte*; Liu, Chao & Chuang, Quart. J. Taiwan Mus. 14: 18, *f. 2-3*. 1961. *pro parte*.

**Phenology**: Flowering in May to June, fruiting in June to July.

**Distribution:** Endemic in Taiwan, only found in Linkou and Nanzhuang in low altitudes, on the open slopes or forest margins.

**Specimens examined:** TAIPEI: Hsinpeitou to Mt. Grass-mountain, alt. 1079 m, Jul. 6, 1960, *M. T. Kao K3616* (TAI); Kuanyinshan, alt. 400–600 m, Jun. 4, 1942, *T. Yoshida s. n.* (TAI); Sanhsia, May 28, 1987, *W. L. Chen* (TAI); Suigenchi, May. 12, 1929, *S. Suzuki s. n.* (TAI); Chihshanyen, Apr. 27, 1933, *K. Mori s. n.* (TAI); Tomitacho, Jun 1, 1932, *Y. Shimada* (NTUF); Waishangchi, alt. 100–300 m, May. 23, 1965, *S. J. Ling s. n.* (TAI). NEW TAIPEI: Linkou, alt. 10–100 m, May 28, 2021, *C. C. Huang 2240* (TAIE); same loc., Jun 28, 2022, *C. C. Huang 2250* (TAIE); same loc., May 29, 2023, *C. C. Huang 2258* (TAIE). HSINCHU: Hsinchu, Jun. 14, 1923, *Y. Simada 1411* (TAI). MIAOLI: Nanzhuang, alt. 150–200 m, May 28, 2021, *C. C. Huang* 2239 (TAIE); same loc., Jun 13, 2022, *C. C. Huang 2250* (TAIE).

**Notes:** *Angelica dahurica* var. *formosana* was poorly known by the local taxonomists of Taiwan due to its extreme rarity. This taxon was categorized into VU (vulnerable) by the Red List of Taiwan Plants (Editorial Committee of the Red List of Taiwan Plants 2017). However, only few “true” *A. dahurica* var. *formosana* were collected during the past hundred year. Most specimens in the herbaria of Taiwan identified as this name are actually *A. pubescens*, especially those collected from Yangmingshan region. Their difference please see the notes under *A. pubescens.*

***3. Angelica hirsutiflora*** Liu, Chao & Chuang, Quart. J. Taiwan Mus. 14(1–2): 19, *pl.2, f. 5.* 1961; Liu & Kao in H. L. Li et al*.*, Fl. Taiwan 3: 940. 1977; Shan & Sheh Fl. Reipubl. Popularis Sin. 55(3): 28. 1992; Kao in T. C. Huang et al*.*, Fl. Taiwan 2nd ed. 3: 1013. 1993; Pan & Watson in Z. H. Wu et al*.*, Fl. China 14: 164. 2005; Seo, Acta Phytotax. Geobot. 56(2): 179. 2005. -TYPE: Taiwan, Shihmen, May 1965, *T. I. Chuang 3979* (Holotype: TAI!).

*Angelica japonica* var. *hirsutiflora* (T. S. Liu, C.Y. Chao & T. I. Chuang) T. Yamazaki, J. Jap. Bot. 65: 222. 1990.

*Peucedanum decursivum auct. non* Maxim.: Henry, List Pl. Form.: 47. 1896; Matsumura & Hayata, Coll. Sci. Univ. Tokyo 22: 173. 1906; Hayata, Icon. Pl. Form. 10: 24. 1921.

*Angelica kiusiana auct. non* Maxim.: Hayata, Gen. Ind. Fl. Form. 32. 1917.

**Phenology:** Flowered in March to April, fruited in April to June.

**Distribution:** In the northern part (New Taipei), Lutao Is. and Dongyin Is., along seashores.

***4. Angelica morii*** Hayata, Icon. Pl. Form. 10: 24, *f.* 15. 1921; Liu, Chao & Chuang, Quart. J. Taiwan Mus. 14(1–2): 19, *f. 4.* 1961; Liu & Kao in H. L. Li et al*.*, Fl. Taiwan 3: 941. 1977; Hiroe, Umbell. World 1410. 1979; Shan & Sheh Fl. Reipubl. Popularis Sin. 55(3): 55, *pl. 25.* 1992; Kao in T. C. Huang et al*.*, Fl. Taiwan 2nd ed. 3: 1013. *pl, 505*. 1993; Pan & Watson in Z. H. Wu et al*.*, Fl. China 14: 167. 2005. -TYPE: Formosa, Mt. Niitaka, Oct. 1906, *U. Mori s. n.* (type: TAIF!).

*Angelica taiwaniana* S. S. Ying, Quart. J. Chin. Forest. 8(4): 125. 1975. –TYPE: Formosa, Daikwanzan to Kwanzan, Jul. 24, 1935, *N. Fukuyama* *s. n.* (Holotype: TAI!)

**Phenology:** Flowering in July to September, fruiting in August to October.

**Distribution:** Endemic in Taiwan, most in central mountains about 2500–3000 m alt. on open slopes, roadsides.

***5. Angelica morrisonicola*** Hayata, J. Coll. Sci. Univ. Tokyo 30: 129. 1911; Liu, Chao & Chuang, Quart. J. Taiwan Mus. 14(1 -2): 20, *f. 3.* 1961; Liu & Kao in H. L. Li et al*.*, Fl. Taiwan 3: 943*.* 1977; Shan & Sheh Fl. Reipubl. Popularis Sin. 55(3): 43. 1992; Kao in T. C. Huang et al*.*, Fl. Taiwan 2nd ed. 3: 1015. 1993; Pan & Watson in Z. H. Wu et al*.*, Fl. China 14: 162. 2005.-TYPE: Formosa, Mt. Niitaka, Nov. 1906, *Kawakami 2129* (Holotype: TAIF!).

*Peucedanum morrisonicola* (Hayata) Hiroe, Umbel. Asia 1: 180, *f. 214.* 1958; Hiroe, Umbell. World 1566. 1979.

*Angelica morrisonicola* var. *nanhutashanensis* Liu, Chao & Chuang, Quart. J. Taiwan Mus. 14(1-2): 21. 1961; Liu & Kao in H. L. Li *et al.*, Fl. Taiwan 3: 943*.* 1977; Shan & Sheh Fl. Reipubl. Popularis Sin. 55(3): 43. 1992; Kao in T. C. Huang *et al.*, Fl. Taiwan 2^nd^ ed. 3: 1015. 1993; Pan & Watson in Z. H. Wu *et al.*, Fl. China 14: 162. 2005. -TYPE: Formosa, Nanhutashan, Jul. 1922, *S. Sasaki* *s. n.* (Holotype: TAI!) syn. nov.

**Phenology:** Flowered in July to September, fruited in August to October.

**Distribution:** Endemic, most in central mountains over 3000 m alt. on open slopes, roadsides.

**Notes:** *Angelica morrisonicola* var. *nanhutashanensis* was described by Liu et al. (1961) who separated it from the typical variety by having smaller leaflet covered with dense hispid on the surface of leaflet. Based on our extensively observation in the field, We found that the leaflet size and hairiness are variable. The plants inhabited in sunny place often have smaller and densely hairy leaflets while those in shady place, such as the scrub of rhododendron and juniper, usually have larger and sparsely hairy leaflets. Moreover, the plants transplanted to the greenhouse display the variation in leaflet size and hairiness. It suggests that these characteristics often varied with the environment and, hence, is not stable for separating taxa. In addition, *A. morrisonicola* var. *nanhutashanensis* exhibited identical cpDNA and nrDNA haplotypes with *A. morrisonicola*, indicating that they cannot be differentiated at the molecular level (see above). Herein, we treat them as a single taxon.

***6. Angelica pubescens*** Maxim, Bull. Acad. St. Petersb. 24: 34. 1878; Hiroe & Constance, Umbell. Japan 107, *f. 55.* 1958; Ohwi, Fl. Japan. Engl. ed. 689. 1965; Hiroe, Umbell. World 1385. 1979.

*Angelica dahurica* (Fisch.) Benth. & Hook. var. *formosana auct. non* (Boiss.) Yen: Liu & Kao in H. L. Li *et al*., Fl. Taiwan 3: 940, *pl. 877*. 1977. *pro minor parte*; Kao in T. C. Huang *et al*., Fl. Taiwan 2nd ed. 3: 1011, *pl, 504*. 1993. *pro minor parte*.

Herbs perennial. Roots thick. Stems 150–250 cm high, usually hollow, green to purple with conspicuously dilated and often bladeless sheaths, slightly velutinous. Leaves 80–100 cm long, 2–3-ternate-pinnate, chartaceous; pinnae elliptical to rhombic, 6–12 cm long, 3–6 cm wide; acute at apex, obtuse or attenuate at base but the ternate pinnae slightly decurrent at base, the margins serrate to doubly serrate, mucronate; reticulate veinlets prominent, velutinous; petioles pubescent. Umbels compound, rays 50–70, about 5–20 cm long, unequal; peduncles scabrescent; bracts 0–2, linear-lanceolate, about 2 cm long; umbellules 5 cm in diameter; pedicels about 40–60; bracteoles 0–4, linear, about 1 cm long. Flowers white calyx-teeth triangular; petals ovate to obovate, with a narrowly inflexed apex; anther purple but yellow in the line of dehiscence; ovary glabrous. Fruit oblong, glabrous, 5–9 mm long, 4–8 mm wide, lateral ribs broadly thin-winged, wider than seed, dorsal ribs filiform. Vitta 3 in the interval, 6–8 in the commissure. Seeds strongly compressed dorsally, inner face plane to concave.

**Phenology:** Flowering in July to September, fruiting in August to October.

**Distribution:** Japan and Taiwan. In Taiwan found in Yangmingshan, Yilan, Hehuanshan and Nengkao in the forest margins from 900 to 3200 m alt.

**Specimens examined:** TAIPEI: Tatunshan, alt. 1092 m, May 28, 1986, *K. C. Yang, 10839* (TAI); Tatungshan, alt. 1060 m, Jun. 11, 1984, *H. Y. Liu 14823* (TAIF); Shilin, alt. 200 m, Jul. 4, 1911, *T. Kawakami s. n.* (TAIF); Sanchih trail to Tatunping, alt. 1040 m, May 6, 1993, *T. Y. Liu 228* (TNU). NEW TAIPEI: Erziping, alt. 900–1000 m, Jul 9, 2021, *C. C. Huang, 2241* (TAIE); same loc., Jun 9, 2022, *C. C. Huang, 2249* (TAIE); same loc., May 15, 2023, *C. C. Huang, 2257* (TAIE). NANTOU: Nengkao, alt. 3260 m, Jul. 14, 1930, *K. Mori 69* (TAI); Tienchih-Yunhai, alt. 2346–2840 m, Sep. 10, 2006, *H. H. Chen 160* (TNU); same loc., Jul. 17, 2007, *H. H. Chen 518* (TNU); same loc., Jul. 15, 2007, *H. H. Chen 570* (TNU); same loc., Jul. 15, 2007, *H. H. Chen 571* (TNU); Yuanfeng, alt. 2700–2800 m, Jul 20, 2022, *C. C. Huang* 2242 (TAIE); Hehuanxi trail, alt. 2500–2600 m, Nov 18, 2021, *C. C. Huan*g *2246* (TAIE). HUALIEN: Hehuan mountain, alt. 2910–2950 m, Nov 16, 2021, *C. C. Huang 2245* (TAIE); Nenggao crossing road, alt. 2700–2800 m, Jul 27, 2022, *C. C. Huang 2252* (TAIE).

**Notes:** In both editions of Flora of Taiwan, this species was misidentified as *A. dahurica* var. *formosana.* It is similar to the latter, but can be distinguished by leaves velutinous (vs. glabrous), stems velutinous (vs. hispid), bracteols 0–4 (vs. 8–10), anther purple mingle with yellow (vs. pure yellow), number of vittae in the fruit interval 3 (vs. 1) and number of vittae in the fruit commissure 8 (vs. 2) (Table 1). *Angelica pubescens* is previously considered to be endemic to Japan. Before us, there is no literature formally documented its presence in Taiwan although its collection can be traced as early as 1911 by Japanese taxonomist. The present study extents its distribution southward to Taiwan.

***7. Angelica tarokoensis*** Hayata, Icon. Pl. Form. 10: 27, *pl. 4, f. 7. & pl. 13, f. 4.* 1921; Masamune, List Vasc. Pl. Taiwan 92. 1954; Liu, Chao & Chuang, Quart. J. Taiwan Mus. 14(1 -2): 21, *f. 7.* 1961; Liu & Kao in H. L. Li et al*.*, Fl. Taiwan 3: 943. 1977; Shan & Sheh Fl. Reipubl. Popularis Sin. 55(3): 43. 1992; Kao in T. C. Huang et al*.*, Fl. Taiwan 2nd ed. 3: 1015. 1993; Pan & Watson in Z. H. Wu et al*.*, Fl. China 14: 162. 2005; –TYPE: Naitaroko, Aug. 1917, *B. Hayata s. n* (Holotype: TAIF!).

**Phenology:** Flowered in June to July, fruited in July to August.

**Distribution:** Endemic in Taiwan, only on limestone wet mountain roadsides, forest margins most in Hualien from 300 to 1800 m alt.

## Conclusions

In this study, we conducted a comprehensive analysis incorporating morphological and molecular characteristics to revise the taxonomic treatments of *Angelica* in Taiwan. As a result of our research, we have revised the classification between *A. dahurica* var. *formosana* and *A. pubescens* and merged two varieties of *A. morrisonicola* into a single taxon. A new taxon, *A. aliensis*, has been identified and found to share a close relationship with *A. tarokoensis*. Based on the morphological and molecular characteristics data, it has been determined that *A. dahurica* var. *formosana*, *A. morrisonicola* and *A. pubescens* should be grouped into the Eurasian *Angelica* clade, while *A. aliensis*, *A. hirsutiflora*, *A. morii* and *A. tarokoensis* should belong to the littoral *Angelica* clade. We provide key to taxa, synopsis, phenology and distribution for each taxon of Taiwan. Our comprehensive analysis of morphological and molecular features has shed light on the taxonomic complexities within *Angelica* in Taiwan, resolving taxonomic issues and providing valuable insights into the phylogenetic relationships of *Angelica* in Taiwan.

### Supplementary Information

**Additional file 1. ** Species, NCBI accession number, and collector number applied in this study.

**Additional file 2. **Alignment, ML tree, and BI tree files of cpDNA.

**Additional file 3. **Alignment, ML tree, and BI tree files of nrDNA.

## Data Availability

All data generated and analyzed during this study are included in this published article and its Additional files.

## Abbreviations

BI: Bayesian inference
CpDNA: *rps16-trnK* Intergenic spacer
EN: Endangered
ETS: External transcribed spacer
HAST: Herbarium, Research Center for Biodiversity, Academia Sinica, Taipei
Hd: Haplotype diversity
MCMC: Markov chain Monte Carlo
ML: Maximum-likelihood
NCBI: National Center for Biotechnology Information
nrDNA: ITS, internal transcribed spacer
Π: Nucleotide diversity
TAI: Herbarium, National Taiwan University
TAIE: Herbarium of Endemic Species Research Institute
TAIF: Herbarium, Taiwan Forestry Research Institute
TNU: Herbarium, National Taiwan Normal University
TNM: TNM Herbarium, Department of Botany, National Museum of Natural Science
VU: Vulnerable
