# Supplementary material for: A taxonomic revision of the genus Angelica (Apiaceae) in Taiwan with a new species A. aliensis
Source: Bot Stud. 2024 Jan 22;65:3. doi: 10.1186/s40529-023-00407-7 (PMC10803708; doi:10.1186/s40529-023-00407-7)
Supplement: Supplementary file 1 — Additional file 1. Species, NCBI accession number, and collector number applied in this study. [file 40529_2023_407_MOESM1_ESM.docx]

Additional File 1. Species, NCBI accession number, and collector number applied in this study.

| species | NCBI accession number | collector number |
| --- | --- | --- |
| cpDNA |  |  |
| *Angelica acutiloba* | KX352468 |  |
|  | NC029391 |  |
| *Angelica aliensis* | OR240204 | Chi-Chun Huang 2244 |
| *Angelica amurensis* | MW436378 |  |
| *Angelica anomala* | NC057139 |  |
| *Angelica apaensis* | NC057132 |  |
| *Angelica biserrata* | MW284876 |  |
|  | MW436379 |  |
|  | NC057127 |  |
| *Angelica cartilaginomarginata* | MT561045 |  |
|  | NC057138 |  |
| *Angelica cartilaginomarginata* var. *foliosa* | MT921959 |  |
| *Angelica dahurica* | MN631000 |  |
|  | MT921972 |  |
|  | MT921974 |  |
|  | MT921978 |  |
|  | OR209144 |  |
|  | OR209145 |  |
|  | OR209150 |  |
|  | OR209151 |  |
|  | OR209153 |  |
|  | OR209156 |  |
|  | OR209157 |  |
|  | OR209159 |  |
|  | OR209161 |  |
|  | OR209162 |  |
| *Angelica dahurica* var. *formosana* | MT921971 |  |
|  | OR209160 |  |
|  | OR240202 | Chi-Chun Huang 2254 |
| *Angelica decursiva* | KT781591 |  |
|  | MW883954 |  |
|  | NC057130 |  |
| *Angelica gigas* | KX118044 |  |
|  | MH793272 |  |
|  | MT921985 |  |
|  | MW900177 |  |
| *Angelica hirsutiflora* | OR240201 | Tsai-Wen Hsu 24169 |
| *Angelica kangdingensis* | NC057131 |  |
| *Angelica keiskei* | NC054219 |  |
| *Angelica laevigata* | MW696157 |  |
| *Angelica laxifoliata* | MT921969 |  |
| *Angelica likiangensis* | NC071789 |  |
| *Angelica megaphylla* | NC057128 |  |
| *Angelica morii* | NC057141 |  |
|  | OR240206 | Chi-Chun Huang 2243 |
|  | OR240209 | Chi-Chun Huang 2262 |
| *Angelica morrisonicola* | OR240210 | Hung-Hsin Chen 100 |
| *Angelica omeiensis* | MW429787 |  |
| *Angelica polymorpha* | MT921961 |  |
|  | NC041580 |  |
| *Angelica pubescens* | OR240203 | Chi-Chun Huang 2257 |
|  | OR240205 | Chi-Chun Huang 2242 |
| *Angelica saxatilis* | MW436381 |  |
| *Angelica shikokiana* | JF279388 |  |
| *Angelica sylvestris* | NC051890 |  |
|  | OM281943 |  |
| *Angelica tarokoensis* | OR240207 | Chi-Chun Huang 2259 |
|  | OR240208 | Chi-Chun Huang 2260 |
| *Angelica tianmuensis* | MW436380 |  |
|  | NC057126 |  |
| *Angelica tsinlingensis* | NC057140 |  |
|  | OM281944 |  |
| *Peucedanum japonicum* | OR240211 | Chi-Chun Huang 2261 |
| nrDNA |  |  |
| *Angelica acutiloba* | AB569093 |  |
|  | AJ131291 |  |
|  | AY548227 |  |
|  | LC773993 |  |
| *Angelica aliensis* | OR242589 | Chi-Chun Huang 2244 |
| *Angelica apaensis* | HQ686375 |  |
|  | HQ686388 |  |
| *Angelica archangelica* | MT735410 |  |
|  | OQ064632 |  |
| *Angelica arguta* | KF619605 |  |
|  | MT735434 |  |
|  | MT735435 |  |
|  | MT735436 |  |
| *Angelica brevicaulis* | MT735417 |  |
|  | GU395170 |  |
| *Angelica callii* | MT735529 |  |
| *Angelica dahurica* | OR242592 | Yu-Tang Zhao 465 |
|  | OR242593 | Quanru Liu 01-1-098 |
|  | OR242596 | Feng Wu 2010036 |
|  | KR052189 |  |
|  | MH711438 |  |
|  | EU418374 |  |
|  | JX022940 |  |
| *Angelica dahurica* var. *formosana* | JX022910 |  |
|  | OR242587 | Chi-Chun Huang 2254 |
|  | OR251501 |  |
| *Angelica dawsonii* | MT735550 |  |
|  | MT735552 |  |
| *Angelica decurrens* | FJ385033 |  |
|  | MT735414 |  |
| *Angelica decursiva* | EU418375 |  |
| *Angelica edulis* | MT735463 |  |
| *Angelica furcijuga* | DQ278164 |  |
|  | LC035465 |  |
| *Angelica gigas* | MT735478 |  |
|  | DQ263575 |  |
|  | JX022913 |  |
|  | KM051435 |  |
|  | MT359958 |  |
|  | MT735477 |  |
| *Angelica hirsutiflora* | OR242586 | Tsai-Wen Hsu 24169 |
|  | MT735570 |  |
|  | MT735571 |  |
| *Angelica japonica* | MH188444 |  |
|  | AY548214 |  |
|  | DQ278166 |  |
|  | MH188441 |  |
| *Angelica kingii* | MT735504 |  |
| *Angelica lineariloba* | MN995840 |  |
|  | MT735442 |  |
| *Angelica lucida* | MT735479 |  |
|  | MT735490 |  |
| *Angelica megaphylla* | JX022934 |  |
| *Angelica morii* | OR242590 | Chi-Chun Huang 2243 |
|  | DQ263573 |  |
|  | DQ263578 |  |
| *Angelica morrisonicola* | OR242595 | Hung-Hsin Chen 100 |
|  | MT735517 |  |
| *Angelica nitida* | JX022927 |  |
| *Angelica pseudoselinum* | JX022933 |  |
| *Angelica pubescens* | OR242588 | Chi-Chun Huang 2257 |
|  | DQ263567 |  |
|  | LC754521 |  |
|  | MT735475 |  |
| *Angelica roseana* | MT735533 |  |
|  | MT735536 |  |
| *Angelica shikokiana* | AB697610 |  |
|  | HQ256682 |  |
|  | LC035466 |  |
| *Angelica stenoloba* | AB697608 |  |
|  | MT735471 |  |
| *Angelica sylvestris* | MT922576 |  |
| *Angelica tarokoensis* | OR242591 | Chi-Chun Huang 2259 |
|  | OR242594 | Chi-Chun Huang 2260 |
|  | MT735564 |  |
| *Angelica tianmuensis* | JX022937 |  |
| *Angelica tomentosa* | MT735446 |  |
| *Angelica triquinata* | MT735458 |  |
| *Angelica tschimganica* | MT735418 |  |
| *Angelica yakusimensis* | MT735472 |  |
| *Peucedanum japonicum* | OR242597 | Chi-Chun Huang 2261 |
